# Clinically Advanced Warty Invasive Squamous Cell Carcinoma of the Cervix with p16 Overexpression—Case Study and Literature Review

**DOI:** 10.3390/reports8040243

**Published:** 2025-11-21

**Authors:** Laura-Andra Petrică, Mariana Deacu, Georgeta Camelia Cozaru, Gabriela Izabela Bălţătescu, Mariana Aşchie

**Affiliations:** 1 “St. Apostol Andrew” Emergency County Hospital, 145 Tomis Blvd., 900591 Constanta, Romania; 2 Faculty of Medicine, Ovidius University of Constanta, 1 Universitatii Alley, 900470 Constanta, Romania; 3 Research Center for the Morphological and Genetic Study in Malignant Pathology (CEDMOG), Ovidius University of Constanța, 145 Tomis Avenue, 900591 Constanta, Romania; 4 Academy of Romanian Scientists, 3 Ilfov Street, 050045 Bucharest, Romania; 5 Institute of Doctoral Studies, Ovidius University of Constanta, 1 Universitatii Street, 900470 Constanta, Romania

**Keywords:** warty (condylomatous) squamous cell carcinoma, cervical cancer, HPV infection, p16 immunostaining

## Abstract

**Background and Clinical Significance:** Warty (condylomatous) squamous cell carcinoma (SCC) of the uterine cervix is a rare papillary variant of SCC, usually associated with good prognosis. **Case Presentation:** We report the clinical case of a postmenopausal woman with vaginal bleeding, anemia, and an enlarged, exophytic tumor mass protruding from the cervix. MRI showed a solid–necrotic cervical–uterine mass with invasion of bladder, rectum, both parametria, and the left ureter, with regional lymphadenopathy and FIGO IVA stage was established. Biopsies from the cervical tumor revealed invasive, well-differentiated SCC with conspicuous koilocytic atypia in superficial and deep nests, consistent with warty (condylomatous) SCC. Immunohistochemistry showed p16 overexpression, an intermediate nuclear proliferation rate, and a non-mutational pattern for p53 immunostaining. Radiotherapy was recommended but the patient’s condition deteriorated rapidly and she died three months after initial diagnosis. Due to the rarity of this type of tumor, we conducted a search on PubMed, Scopus, and Web of Science from inception to 31 July 2025 and we identified ten reports available for evaluation. A total of 32 cases were identified, usually with FIGO stage I or II, mostly with low-risk HPV infection and with good prognosis. **Conclusions:** The advanced stage and limited tolerance for therapy in this case emphasize the importance of HPV vaccination and HPV-based screening to prevent late, non-curable presentations. Accurate distinction from condyloma acuminatum and verrucous or papillary SCC is clinically relevant because management and outcomes differ. Since some of the cases reported in the literature had a worse clinical course, with shorter disease-free survival and overall survival, including our case, further research is mandatory in the future to unravel those features which might predict a poor outcome.

## 1. Introduction and Clinical Significance

On the global scale, uterine cervix cancer continues to be a major health problem and represents the 4th cause of female cancer with an incidence of 14.1%, after breast, lung, and colorectal carcinoma [1]. Prevention programs and different therapeutic options are available, but there are countries or different regions where these solutions are not applicable.

It mostly affects women younger than 45 years old [2] and is responsible for 7.1% of deaths of women worldwide, being an important cause of mortality in 37 countries [1,2]. There is a high variability between countries regarding incidence and mortality, up to 10 times, with peaks in those countries with the lowest Human Development Index (HDI), an instrument used to classify countries into different levels of development based on life expectancy, education, and income per capita [2,3]. In Romania, the age-standardized incidence and mortality rates for cervical cancer are approximately 22.6 and 10.8 per 100,000 women, respectively, among the highest in the European Union [1].

Squamous cell carcinoma (SCC) of the cervix is a prevalent form of cervical cancer and its main etiopathogenic factor is infection with human papillomavirus (HPV), the majority of cases are due to infection with high-risk HPV types 16 and 18 [4,5]. According to its presence or absence, the 2020 World Health Organization classified SCC of female genital tumors in HPV-associated (HPVA) and HPV-independent (HPVI) tumors [4,5].

Morphological subtypes of SCC (keratinizing, non-keratinizing, papillary, basaloid, warty/condylomatous, verrucous, squamo-transitional, and lymphoepithelial-like), which were included in the 2014 WHO classification [6], are no longer required to be reported according to WHO 2020 quidelines [4]. Nevertheless, identifying and correctly reporting the morphological subtypes of SCC of the uterine cervix is very important, especially for future research and is still part of the 2022 International Collaboration on Cancer Reporting (ICCR) guidelines [7].

Warty or condylomatous carcinoma is a rare and specific type of SCC, which initially was described in the vulvar region, but also in other locations like oral mucosa, anal region, vagina, urinal bladder, and penis [8,9]. In 1992, Kurman RJ et al. (1993) first recognized this type of tumor in the uterine cervix [10]. Only a few cases of warty SCC of the cervix have been reported, having a frequency of 1.9–2.2% reported by the research of Yordanov et al. (2018) [11,12].

It is defined as a malignant tumor with a biphasic component: a superficial component with papillary architecture and morphological features like condyloma acuminatum or a Bowenoid lesion of the vulva [4,6]; an invasive, deeper component represented by invasive squamous cell carcinoma, with or without keratinization, usually with a well or moderate differentiation [11,13]. The name “warty” or “condylomatous” described the exophytic component of the tumor which usually is large with “wart-like” or “feather-like” aspects [9]. This gross appearance represents a frequent source of confusion with other SCC with papillary patterns of growth but is different in terms of prognosis and clinical management [9].

In the present study, we report a rare case of an advanced and aggressive invasive squamous cell carcinoma of the cervix with warty subtype that is HPV-dependent with p16 overexpression. The severe clinical evolution of our case is in contradiction with its usually good associated prognosis, highlighting the importance of a correct and early diagnosis of cervical cancer through screening programs and the important role of HPV vaccination as a prevention measure.

## 2. Case Presentation

We present the clinical case of a postmenopausal 67-year-old female patient from rural area, who subsequently sought medical assistance at the Emergency Department of the “Saint Apostle Andrew” Constanta County Emergency Clinical Hospital, presenting with an alarming occurrence of vaginal bleeding that had recently manifested, accompanied by a significant sense of weakness and episodes of dizziness.

The patient was admitted to the Clinical Obstetrics and Gynecology Department II and the following procedures were performed: gynecological and rectal examination; magnetic resonance imaging (MRI) of the abdomen and pelvis (native and with contrast); cystoscopy; and cervical biopsy for morphological and immunohistochemical evaluation.

Immunohistochemical evaluation was performed on four µm-thick sections of a formalin-fixed, paraffin-embedded tissue block. After the epitope retrieval, tissue sections were incubated with the following antibodies from Biocare Medical (ready-to-use): p16 (INK4a); Ki67 (SP6 clone); and p53 (DO-7 clone). The chromogen used was 3,3′ diaminobenzidine (DAB), with brown staining and then Mayer’s hematoxylin was used to counterstain the sections.

Microscopic and immunohistochemical slides were scanned with a HuronTISSUEScope LE120 scanner (Huron Technologies International Inc., St. Jacobs, Ontario, Canada.), then they were examined and photos were taken.

**Figure 1:**
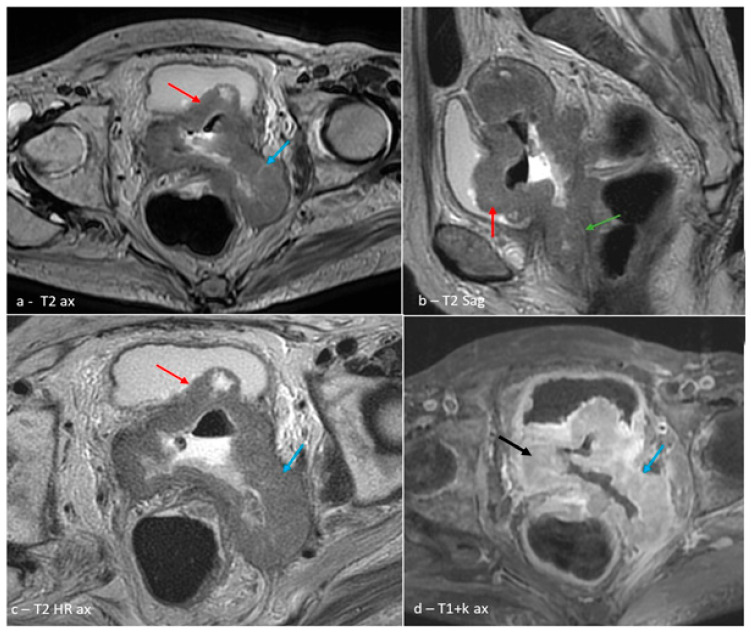
Magnetic resonance imaging (MRI) of the abdomen and pelvis. Solid-necrotic heterogeneously enhancing cervical–uterine mass invading the urinary bladder ((**a**–**c**)—red arrows), upper third of the vagina ((**b**)—green arrow), and with mainly left parametrical involvement ((**c**,**d**)—blue arrows; black arrow—tumor mass).

Gynecological examination of the patient revealed an enlarged, exophytic tumor mass with irregular surface, focal area of ulceration, and active bleeding. Imaging studies described an expansive–infiltrative cervical–uterine mass with bladder, rectal, bilateral parametrial, and left ureteral invasion, associated with retroperitoneal and sub peritoneal lymphadenopathy (Figure 1a–d). Blood analysis revealed anemia (hemoglobin level: 9.1 g/dL) with no other notable hematologic abnormalities.

**Figure 2:**
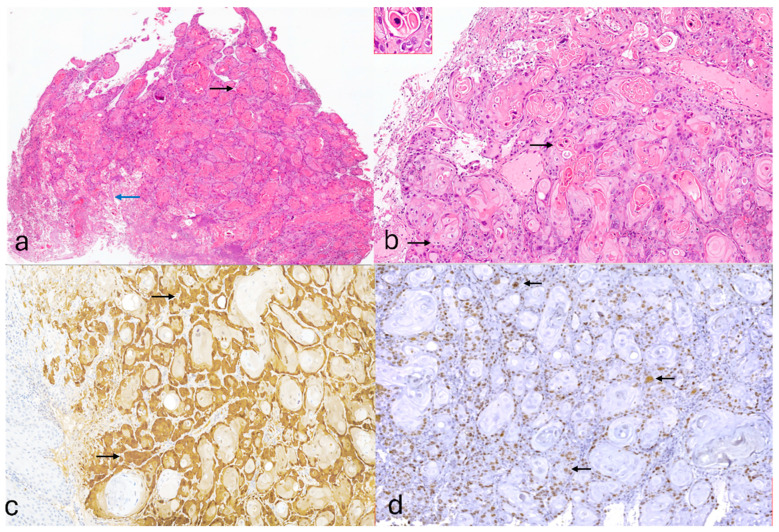
(**a**) Warty SCC exhibits an irregular verruciform (wart-like) architecture, with angulated tumor nests (blue arrow) of well differentiated squamous cell carcinoma (black arrow) infiltrating the underlying stromal tissue (col HE, ob. 20×); (**b**) the invasive component is represented by well-differentiated squamous cell carcinoma with frequent atypical koilocytes (black arrow) and dyskeratosis (in case) (col HE, ob. 100×; in case ob. 200×); (**c**) immunostaining with p16 antibody revealed an intense and diffuse positive reaction (black arrow)—in “block-pattern” (IHC, ob. 100×); (**d**) diffuse positive nuclear stain of Ki67 antibody (black arrow) in the invasive SCC component (IHC, ob. 100×).

Histopathological examination of the cervical biopsy described an epithelial malignant proliferation suggestive for invasive squamous cell carcinoma; it was well differentiated and associated with a focal lesion of high-grade intraepithelial lesion. Conspicuous and diffusely nuclear koilocytic-type atypia were identified in both superficial and deeper portions of the tumor, a morphological feature characteristic for warty-type SCC (Figure 2a,b).

The immunohistochemical interpretation revealed the following: positive nuclear immunostaining for p16 which was diffusely and “block-pattern” positive (Figure 2c); positive nuclear staining for Ki67 in 30% tumoral cells (Figure 2d); and a focal nuclear positive immunostaining for p53—“wild-type” pattern.

Clinical and paraclinical evaluation established the diagnosis of invasive squamous cell carcinoma of uterine cervix, with a warty-pattern, which was well differentiated and HPV-dependent, stage IVA. According to the present national guidelines, oncological management included palliative radiotherapy. Due to worsening anemia during hospitalization, she required correction through a transfusion of one unit of iso-group, iso-Rh red blood cell mass. Unfortunately, the general condition of the patient worsened and she passed away approximately three months after initial diagnosis.

## 3. Discussion

### Literature Review

The literature review was performed using a PICO tool [14]: P (patient/population/problem)—women diagnosed with warty (condylomatous) squamous cell carcinoma of the cervix; I (intervention/exposure/risk factor)—histopathological diagnosis and clinical management of warty SCC; C (comparison)—conventional squamous cell carcinoma of the cervix or other histological variants; O (outcome)—clinical presentation, HPV association, treatment response, and outcome.

We included PubMed, Scopus, and Web of Science as electronic databases, and the search period was from the inception of each database until 31 July 2025. In accordance with the Preferred Reporting Items for Systematic Reviews and Meta-Analyses (PRISMA) 2020 guidelines [15], we used the following key words: “warty” or “condylomatous” and “invasive squamous cell carcinoma” and “cervix”. Reference lists of relevant articles were also screened by the “Backward Citation Chaining” method to identify additional sources and relevant works.

Eligibility criteria included original studies reporting cases of warty (condylomatous) squamous cell carcinoma of the uterine cervix, irrespective of study design (case reports, case series, and retrospective studies) and studies that were published in the English language. Reviews, studies referring to other subtypes of SCC, studies without specifying histological subtype, or those not consistent with our subject of study were excluded.

**Figure 3:**
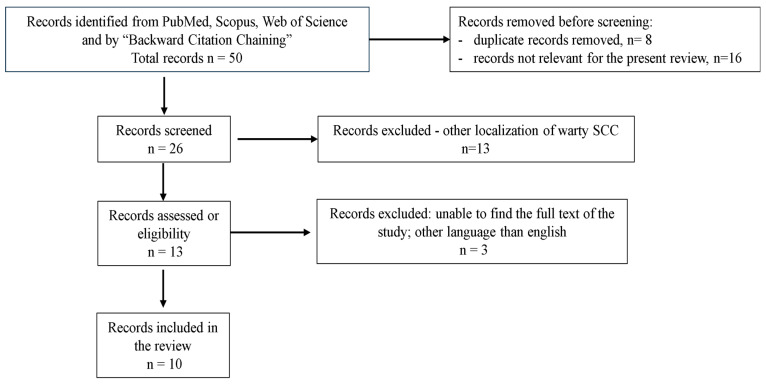
Flow chart of the literature review process—PRISMA diagram.

The literature search and study selection process are shown in the flowchart of the systematic review (Figure 3). Of 50 records, 10 articles were suitable for review (Table 1). A total of 32 cases were identified, usually post- or perimenopausal women and mostly with FIGO stage I or II. HPV infection was identified in all cases, usually with low-risk HPV genotypes, (6/11 and 16/18/33), but also high-risk HPV genotypes (16, 18); few cases were attributed to infection with Epstein–Barr virus (EBV) or had a co-infection with HPV. A negative result for p16 immunostain was recorded in many of the cases and was associated with a good prognosis. Nevertheless, few cases had a worse outcome with shorter disease-free survival and overall survival, which does not fit within the broader context.

Among the 32 WSCC cases identified across the reviewed literature, the majority presented at early FIGO stages (I or II). For example, in the large cohort reported by Yordanov et al. [11,12], 14 cases were reported with either IB1 or IB2 staging, all with favorable outcomes and no disease recurrence during long-term follow-up (ranging from 4 to 115 months). Similar findings were observed in studies by Cho et al. [16] and Ng et al. [17], where early stage tumors exhibited indolent behavior and responded well to standard treatment.

While WSCC is typically associated with low-risk HPV types (especially HPV-6 and HPV-11), these are not always predictive of favorable outcomes. Masuda et al. [18] reported a patient with stage IB1 WSCC, HPV-6 positivity, and negative p16 expression who developed rapid lymphatic and pulmonary metastases within 3 months of diagnosis. Similarly, Rokutan-Kurata et al. [19] described a T1bN1M0 case in a younger patient with HPV-6 infection and an unfavorable clinical course, resulting in death within 22 months. These cases highlight that low-risk HPV genotypes may occasionally be associated with unexpectedly aggressive tumor behavior, challenging the assumption that genotype alone predicts prognosis.

The expression of p16 (INK4a) is considered a surrogate marker for oncogenic HPV activity. In our literature review, however, p16 expression was negative in many cases, particularly those with low-risk HPV genotypes [11,12,16,20]. Conversely, the only other case with p16 overexpression reported by Landeyro et al. [21] resulted in a fatal postoperative complication.

One of the most striking patterns observed is the mismatch between the well-differentiated, “low-grade” morphology of WSCC and its occasional aggressive clinical course. Histologically, WSCC is characterized by papillary architecture, koilocytotic atypia, and minimal invasion, features typically associated with good prognosis [5,9,12].

Cervical cancer remains a major public-health issue. The World Health Organization estimates that over 600,000 women were diagnosed with cervical cancer and 342,000 died in 2020 [24]. A correct diagnosis is very important both for determining prognosis and for evaluating therapeutic options.

Persistent infection with high-risk HPV types accounts for almost 95% [24] or up to 99% of cervical cancers, according to other research [25]; nevertheless, other risk factors where identified by recent studies and proved that they can play an important role in the carcinogenesis of SCC: infection with human herpesvirus II or with cytomegalovirus [26]; infection with Epstein–Barr virus (EBV) [27]; sexually transmitted infections (human deficiency virus—HIV and *Chlamydia trachomatis*) [2]; long-term oral contraceptive or tabaco use [2]; and poorly developed countries [2,3,4].

Genital HPVs are divided into low-risk types (e.g., 6 and 11) that produce warts and high-risk types (e.g., 16, 18, 31, 33, 45, 52, and others) that can cause precancerous intraepithelial lesions that if persistent may lead to cancer [28]. Although most infections clear spontaneously within one to two years, a small fraction of high-risk infections persist and act as cancer precursors [28]. Several cofactors like early sexual activity, multiple partners, smoking, other sexually transmitted infections, and immunosuppression facilitate persistent infection and progression [24].

Persistent high-risk HPV infection is responsible for nearly all cases of cervical cancer. Approximately 95% of cervical malignancies occur in women who do not receive appropriate clinical follow-up or treatment for HPV-induced precancerous lesions [29]. Progression to invasive cancer can accelerate from the usual 15–20 years to just 5–10 years in immunocompromised patients [29]. From all high-risk types, HPV-16 and HPV-18 together account for about 70% of global cervical cancers, underscoring the importance of preventive vaccination and screening high-risk types [29].

Although most cervical SCCs are of the keratinizing or non-keratinizing type, uncommon variants such as warty (condylomatous) SCC pose diagnostic challenges, especially on tumor biopsies. Warty SCC is a rare subtype of SCC and is an HPV-induced tumor, firstly described in the vaginal or anal region. Reported genotypes in cervical WSCC include high-risk HPV (HPV-16, HPV-18, and HPV-33) or low-risk HPV (HPV-6 and HPV-11) but sometimes there are mixed co-infections within the same tumor [16,20,22]. The study of Yordanov AD et al. (2020) proved an infection of EBV in four cases of warty SCC from eleven cases examined by PCR, two of them being co-infection with HPV [12]. Because many cases proved to be due to low-risk HPV types, these tumors sometimes are referred as “low-risk HPV-associated well-differentiated SCC of the cervix with koilocytotic morphology” spreading more confusion around the terminology of this type of squamous cell carcinoma [23].

It is a tumor with papillary architecture usually described as a hybrid feature of condyloma, with a papillary or feather-like surface rich in atypical koilocytosis overlying an invasive squamous carcinoma component, which can be well or moderately differentiated [12,16]. Sometimes malignant foci seem to emerge as a direct transformation from the condylomatous component, which indicates a significant correlation between these two pathological entities [11,16]. Another morpho-pathological feature is the presence of an irregular and jagged interface that exists between the tumor and the surrounding stroma [9,30]. Cytologically, viral infection induces koilocytic changes in the infected cell characterized by the presence of nuclear atypia, a high nuclear/cytoplasmic ratio, perinuclear cytoplasmic halos, dyskeratosis, or multinucleation [17], features that were also identified in our case.

Because of their papillary exophytic pattern of growth, warty SCC must be differentiated from other lesions, benign or malignant, with similar architecture like condyloma acuminatum, verrucous carcinoma, papillary squamous cell carcinoma, or squamous-transitional carcinoma [21,31]. Warty SCC of the cervix is usually a slow-growing tumor but, in contrast to condyloma acuminatum (wart) or verrucous carcinoma, presents a potential for regional metastasis [9]. Consequently, it is imperative to distinguish it from other verruciform neoplasms and to establish the correct diagnosis.

Condyloma acuminatum (wart) is a benign type of tumor and like warty SCC is an HPV-induced tumor, but HPV type 6 and 11 which also cause koilocytic viral cytopathic changes can be involved [22]. It is also differentiated from warty SCC through its rounded papillae, prominent fibrovascular cores, and by lacking stromal invasion and cytologic atypia [31]; the presence of significant atypia and of atypical mitoses in any condyloma (that is architecturally typical) should raise the suspicion of warty carcinoma. Giant condyloma acuminatum is its larger counterpart with a cauliflower-like aspect, locally destructive mass that compresses rather than invades tissue and shows mild hyperkeratosis with abundant koilocytes [31].

The most important differential diagnosis is with verrucous carcinoma, which can easily be confused with warty SCC, especially on small biopsy samples, because it also has an exophytic component with papillary architecture, but it forms coarse, firm papillae with a bulbous base and thick parakeratosis and do not have fibrovascular cores or koilocytes in the proliferative epithelium [12]. The presence of nuclear koilocytic atypia represents one of the most important key features in differentiating warty SCC from verrucous carcinoma, these being frequently identified in both superficial and deep infiltrative components in warty SCC [5,10], as we encountered in our case. Usually, verrucous carcinoma expands to the surrounding tissue by “pushing” edges, in contrast to warty SCC which has an irregular or a diffuse invasion margin [32]. Unlike verrucous carcinoma, warty SCC exhibits more numerous, often abnormal mitotic figures and lesions adjacent to the tumor frequently show cervical intraepithelial neoplasia with warty or basaloid characteristics [22], as we also observed in our case. Furthermore, verrucous carcinoma has an excellent prognosis, with rare metastasis [22] compared to warty SCC which, even if it has a slow growth, is still associated with higher risk of distant metastasis [9].

It is also very important to distinguish verrucous carcinoma from wart SCC because treatment ranges from conservative management to radical surgery or chemoradiation. Surgical excision is the first-line treatment for verrucous carcinoma since radiotherapy can induce anaplastic transformation and metastasis [33]. On the other hand, therapeutic management for warty SCC is more complex and includes radical hysterectomy plus pelvic lymphadenectomy and radiotherapy, sometimes chemotherapy is also necessary [22]. A correct distinction between these two types of cancer is essential to avoid overtreatment [13].

Another possible differential diagnosis is with papillary SCC or squamo-transitional carcinoma of the cervix, where its papillary structures are lined by a transitional type of epithelium with atypical cells but lacking koilocytes [20]. The clear cell of this type of SCC can be distinguished from koilocytic changes by their resemblance with urothelial cells and their more uniform nuclei [17].

Immunohistochemistry can be a useful tool in establishing diagnosis. Diffuse and strong p16-INK4a staining is consistently present in almost all HPV-associated cancers and is frequently used as a surrogate marker for HPV infection [4]. In the meta-analysis study performed by Huang K et al. (2014), it was proven that the patients with cervical cancer associated with overexpression of p16 have a better chance of disease-free survival [34]. Distinguishing HPV-dependent cervical cancers from those HPV-independent, which are rare, has a prognostic value since the last one is associated with a worse outcome [35]. HPV-independent cancers tend to be more frequently associated with an advance stage or lymph node metastasis [36]. Searching the literature, the vast majority are negative for p16 staining. For instance, Yordanov et al. (2018, 2022) reported that most of their cases were p16-negative and had favorable outcomes despite their association with high-risk HPV or EBV co-infection [11,12]. Similarly, Cho et al. (1988) [16] and Kim et al. (2017) [20] described HPV-positive tumors with absent p16 expression [16,20]. In the present case we obtained strong, block-typep16 immunostain, which indicates HPV-induces carcinogenesis, but the clinical prognosis was poor. There is only one other case with FIGO clinical stage IIA and with p16 overexpression which had a poor clinical outcome, suggesting that there are other negative factors involved in its severe evolution [21].

HPV genotyping was not possible in the present case and this represents a limitation for our study. Its overexpression is associated with the presence of infection with an HPV type but did not distinguish between high-risk and low-risk HPV genotypes. This differentiation is crucial, as the recent literature has documented instances of WSCC associated with low-risk types (e.g., HPV-6 or HPV-11) that nonetheless exhibited aggressive clinical characteristics, including early metastasis and rapid progression [18,19]. The specific HPV genotype could have yielded enhanced insight into the oncogenic mechanisms that underlie the aggressive trajectory observed in our patient.

Ki-67 is a proliferation biomarker and a predictive factor for tumor development; it labels the nuclei of dividing cells [36]. Although Ki-67 biomarkers have been extensively studied in precursor lesions (often together with p16INK4a), relatively fewer and heterogeneous data exist for invasive cervical carcinoma; nevertheless, meta-analyses show that a higher Ki-67/MIB-1 index is associated with worse overall survival in cervical cancer [37]. Ki-67 also has predictive value: measuring the Ki-67 index before and after therapy (e.g., chemoradiation or chemotherapy) can help assess treatment response, since a significant post-treatment decrease in Ki-67 has been correlated with therapeutic response in several studies [38,39]. Ng et al. [17] and Olaru et al. [22] reported moderate Ki-67 expression consistent with well-differentiated tumor morphology. In our case, we noticed an intermediate proliferation rate in contrast with the clinical stage or with its aggressive clinical evolution, indicating that proliferation rate alone may not be a reliable predictor of aggressiveness in WSCC, especially when there is small biopsy sample from the superficial part of the tumor, which may underestimate the tumor’s true biology.

The p53 biomarker is important to evaluate because p53 oncoprotein controls cell division and apoptosis and this why it is the “guardian of the genome” [40]. According to the research by Petijean A et al. (2007), p53 mutations are identified in almost all types of cancer, in percentages ranging from 5 to 50% [41]. In cervical cancer, p53 is inactivated by degradation via complex formation with the human papillomavirus (HPV) oncoprotein E6 and E6-associated protein (E6AP), an E3 ubiquitin-protein ligase [42].

According to Chan MP et al. (2019), p53 immunostaining might support the involvement of HPV infection when its expression is “wild-type”—a normal pattern (focal positive nuclei without diffuse overexpression) [13]. An abnormal p53 immunostain, which can be “null-type” (no positive cell) or p53 overexpression (intense and diffuse positive cell) correlates with TP53 mutation and should trigger a search for HPV-independent biology [5]. In the present study, the p53 immunostain was “wild-type” and together with p16 overexpression support the HPV-related biology. A similar result was obtained for p53 staining in the case reported by Ates D et al. (2024) with FIGO IVA stage but with negative p16 staining [23], proving that this type of cancer must be further studied to unravel all its molecular changes to be able to better establish the prognosis.

In the last 15 years, the Romanian Society of Obstetrics and Gynecology (SOGR) has published four clinical guidelines on cervical cancer (2010, 2019, 2022, and 2024) and FIGO revised the cervical cancer staging system in 2018. The 2018 revision introduced imaging and pathology to assign stage and created new sub-categories for stage IB and lymph-node-positive disease, but stage IV remained unchanged in its definition [41]. Under both FIGO 2009 and 2018 systems, stage IVA describes a tumor that has extended into adjacent pelvic organs, typically the bladder or rectum and stage IVB includes spread to distant organs (lung, bone, or distant nodes) [43].

Nevertheless, the therapeutic approach for stage IV disease has not changed and because it represents locally advanced or metastatic disease, primary surgical treatment is generally excluded. International guidelines such as the US National Cancer Institute and the National Comprehensive Cancer Network (NCCN) recommend concurrent chemoradiation (external beam radiation and brachytherapy) with weekly cisplatin or carboplatin for stage IIB to IVA disease [44,45]. Recent meta-analyses have shown that adding cisplatin to radiotherapy improves overall and disease-free survival compared with radiation alone [45]. While pelvic exenteration may be considered in highly selected patients with isolated organ invasion and no metastases, such cases are uncommon; the general rule is to avoid primary surgery and deliver definitive chemoradiation. For stage IVB, therapeutic options are limited to palliative and systemic treatments [44]. In our case, the patient presented with an advanced disease (FIGO IVA), indicating invasion of adjacent pelvic organs and ineligibility for primary surgery and her treatment option consisted of radiotherapy. Despite initiation of radiation with palliative intent, her biologic condition deteriorated rapidly and she succumbed after only 3 months from initial diagnosis, even if this morphological subtype of SCC is usually associated with a good prognosis [11,12,16,17]. The observed aggressiveness may be attributable to the advanced clinical stage; however, two additional cases reported in the literature demonstrate poor outcomes even in the absence of advanced stage disease. Masuda M et al. (2018) described a postmenopausal woman with FIGO stage IB1 and a low-risk HPV (HPV-6) infection and negative p16 immunostaining, who experienced an aggressive clinical course characterized by multiple lymph node and lung metastases within three months of the initial diagnosis [18]. Similarly, Rokutan-Kurata M et al. (2020) reported a premenopausal woman with T1bN1M0 disease who succumbed to the disease after 22 months [19]. These findings underscore the need for close surveillance in all cases of this morphological subtype of SCC, given the potential for rapid disease progression.

## 4. Conclusions

Warty SCC is a special morphological subtype of invasive SCC of the cervix usually associated with good prognosis. Rarely, it has an unfavorable outcome as it was observed in our case. Its advance clinical stage identified at initial diagnosis limited the therapeutic options. These results emphasis the importance of the screening programs which must be performed not only in the urban areas but also in the rural regions.

In addition to public health measures, our findings highlight the importance of accurate pathological evaluation. Pathologists should maintain a high index of suspicion when encountering papillary or exophytic cervical lesions, especially those with koilocytic atypia, and should include WSCC in the differential diagnosis. Ancillary tests with p16, ki67, and p53 biomarkers sustain the role of HPV infection and helps the pathologist to provide a correct diagnosis and to estimate the prognosis.

More than that, we once again underscore the importance of HPV testing which promotes effective detection and surveillance and its integration into screening programs has the potential to reduce incidence and mortality.

Understanding the biology of this type of cancer is essential for developing effective prevention strategies, including vaccination against HPV and promoting regular screening to detect precancerous changes early.

## Figures and Tables

**Table 1:** Clinicopathological features of the cases included in the scientific records selected for the literature review.

Study	Country	Nr	Age (y.o)	Clinical Stage	P16 IHC	HPV ISH	EBV ISH	PCR	Outcome	Follow-Up
Yordanov A (2018, 2022) [11,12]	Bulgaria	14	Mean 48 range 29–72	FIGO IB1 (*n* = 6) FIGO IB2 (*n* = 8)	-	1 case + * (HR- HPV)	1 case +	HR-HPV poz, (*n*= 2) EBV poz (*n* = 2) HR-HPV+ EB1 poz (*n* = 2) *	Favorable	minimum 4 maximum 115 months
Cho NH (1988) [16]	Korea	9	Mean 54,1 range 44–77	IA1-IIA	-	6 + 11 (*n* = 1) 33 (*n* = 1) 16 (*n* = 7)	-	-	Favorable	-
Ng WK (2002) [17]	China	3	50, 69, 81	IB (*n* = 2) IIA (*n* = 1)	-	-	-	-	Favorable	-
Masuda M (2018) [18]	Japan	1	43	FIGO IB1	neg	-	-	HPV6	Unfavorable	12 months DFS ^!^—1 month
Rokutan-Kurata M (2020) [19]	Japan	1 ^☼^	43	T1bN1M0	-	6	neg	-	Unfavorable	DFS ^❄^—10 months OS—22 months
Kim HJ (2017) [20]	Korea	1	75	FIGO IB1	neg	6 + 42	-	HPV6 HPV42	Favorable	follow-up: 3 months
Landeyro J (2011) [21]	Spain	1	73	FIGO IIA	poz	no	-	HPV16	Unfavorable	patient died after surgery from postoperative bleeding complications
Olaru OG (2020) [22]	Romania	1	58	FIGO IIB	neg	-	-	HPV45	Favorable	5 years
Ates D (2024) [23]	Turky	1	30	FIGO IVA	neg	6 + 11	-	-	Favorable	36 months

HPV—human papilloma virus, HR-HPV—high risk HPV; EBV—Epstein–Barr virus, ISH—in situ hybridization, PCR—polymerase chain reaction, OS—overall survival; DFS—disease free survival; poz—positive; neg—negative * 11 cases evaluated; ^☼^—case nr three, from a series of five cases with low-risk SCC of the cervix; ^!^ after 1 month—multiple supraclavicular, mediastinal, and para-aortic lymph node; after 3 months—lung metastases, ^❄^ lung metastases.

## Data Availability

The original data presented in the study are included in the article, further inquiries can be directed to the corresponding author.
